# What Does “Palliative” Mean? Sentiment, Knowledge, and Public Perception Concerning Palliative Care on the Internet since the COVID-19 Pandemic

**DOI:** 10.1089/pmr.2024.0057

**Published:** 2024-12-04

**Authors:** Joachim Peters, Maria Heckel, Eva Breindl, Christoph Ostgathe

**Affiliations:** ^1^ Department of Palliative care, University Hospital, Friedrich-Alexander-Universität Erlangen-Nürnberg (FAU), Erlangen, Germany.; ^2^ Chair of German Linguistics, Friedrich-Alexander-Universität Erlangen-Nürnberg (FAU), Erlangen, Germany.

**Keywords:** COVID-19, language analysis, linguistics, palliative care, public opinion, social media

## Abstract

**Background:**

Little is known about the public perception of palliative care during and after the pandemic. Assuming that analyzing online language data has the potential to collect real-time public opinions, an analysis of large online datasets can be beneficial to guide future policymaking.

**Objectives:**

To identify long-term effects of the COVID-19 pandemic on the public perception of palliative care and palliative care-related misconceptions on the Internet (worldwide) through natural language processing (NLP).

**Design:**

Using large language model NLP analysis, we identified public attitudes, opinions, sentiment, and misconceptions about palliative care on the Internet, comparing a corpus of English-language web texts and X-posts (“tweets”) (02/2020–02/2022) with similar samples before (02/2018–02/2020) and after the pandemic (03/2022–02/2024).

**Setting:**

The study is a statistical analysis of website and social media data, conducted on six large language corpora.

**Results:**

Since the COVID-19 pandemic, palliative care situations are more often portrayed as frightening, uncertain, and stressful, misconceptions about the activities and aims of palliative care occur on average 44% more frequently, especially on the social media platform X.

**Conclusions:**

The impact of the COVID-19 pandemic on public discussion on social media continues to persist even in 2024. Insights from online NLP analysis helped to determine the image of palliative care in the Internet discourse and can help find ways to react to certain trends such as the spread of negative attitudes and misconceptions.

## Key Statement

Analyzing large quantities of social media data, our study adds important insight into the long-term consequences of the COVID-19 pandemic on the public perception of palliative care, including mostly negative emotional attitudes and a higher occurrence of misconceptions regarding palliative care situations. The present work was performed by Joachim Peters in (partial) fulfillment of the requirements for obtaining the degree “Dr. rer. biol. hum.” at the Friedrich-Alexander-Universität Erlangen-Nürnberg (FAU).

## Background 

Palliative care is a holistic, person-centered field of medical care that is committed to specific ethical standards and aims to control the symptoms of serious illnesses at the end of life and to improve the quality of life of patients, their families, and loved ones.^1,2^ Key concepts of palliative care can be observed in internal communication, in communication toward patients, and in public relations.^3^ Palliative care and its objectives are subject to public discourses, e.g., in social media or websites. Since the COVID-19 pandemic meant a serious disruption for both patients and the health care system, it is of interest whether changes in the public perception of palliative care since the beginning of the pandemic situation can be observed.

The significant effects of the COVID-19 pandemic on palliative care and palliative medicine on caregivers, patients, families, and media have been examined using both qualitative^4^ and quantitative approaches.^5–7^ Research has identified mostly negative consequences of the pandemic for patient well-being, e.g., it was referred to as a “tsunami of suffering.”^7,8^ Multimorbid older adults in particular were exposed to the severe consequences of COVID-19 symptoms, for example, in the form of shortness of breath, restricted mobility, psychological and psychosocial stress, restlessness, depression, or loneliness as a result of a lack of social contacts.^8–10^ Palliative care plays a key role in the relief of suffering by offering symptom management, communication, care coordination, and emotional support, also in crisis situations.^11^

Misconceptions concerning the objectives of palliative care still present obstacles to optimal care.^12,13^ We understand misconceptions as false, persistent subjective beliefs that typically originate from informal sources.^14^ Misconceptions can have negative effects on the understanding of complex phenomena, but also influence behavioral decisions. Health literacy is an important concept to avoid the formation of misconceptions and to reduce existing misconceptions.^15^ Previous investigations into misconceptions were largely based on a survey approach, not on empirical analyses of discourses.^16^ In many countries, broad sections of the population have little or no knowledge of palliative care, and the word *palliative* can have a negative impact on the perception of a treatment situation, although information is provided through publicly available brochures or websites.^3,17,18^ The COVID-19 pandemic has shown how quickly information and opinions spread on the Internet and how information and certain metaphors can affect people’s behavior.^19–21^ Therefore, it is important to examine the discourses about palliative care that are conducted on social media platforms. The effects of the COVID-19 pandemic with regard to changes in the mental health, coping strategies, and trust in medicine during the pandemic have been examined in several studies in which the perspectives of patients and relatives were considered.^20^ However, from an empirical perspective, it has not yet been clarified what long-term effects the pandemic has had on perceptions and possible misconceptions concerning palliative care.

### Aim

The aim of our study was to extract changes in public perception of palliative care in online discourses *during* and *after* the COVID-19 pandemic using large datasets of natural language.

### Design

Natural language processing (NLP) is increasingly being used in palliative care research because analyzes based on language data can provide an empirical basis for the observation of discourse changes.^5,6,21,22^ We performed a combined study consisting of quantitative and qualitative corpus linguistic methods. Corpus linguistics is an empirical method for the study of language by way of a text corpus, a large collection of texts with certain characteristics. Statistical methods, e.g., effect size measures and significance testing were used to gain insights into the characteristics of news and social media posts about palliative care.^22^ In order to get an overview of the public perception of palliative care, we aimed to make the largest possible section of the online discourse available for analysis.^23^

### Setting

**FIG. 1.**
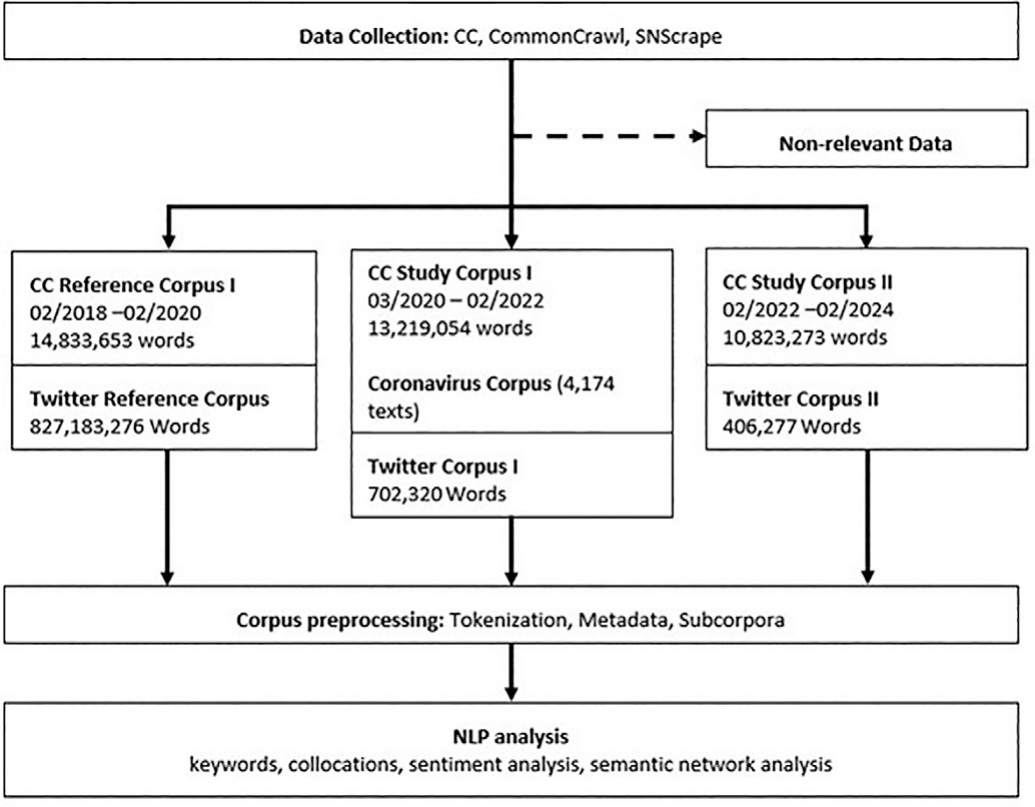
Schematic procedure for data collection and corpus preprocessing.

Three corpora were used (Fig. 1): (1) The Coronavirus Corpus (1.5 billion words, 2/2020–12/2022) was used for an overview of the situation in online media. It is designed to be “the definitive record of the social, cultural, and economic impact of the COVID-19 pandemic.”^24^ (2) A separate corpus of web texts (2/2020–2/2024) was used to capture the public perception on the Internet. It was created with the strings *palliat* and *end*of*life*, using data from the CommonCrawl corpus,^25^ consisting of English-language websites with the ccTLD-domains .com, .org and .uk. Twenty-three, 219 texts with relevant thematic focus remained in the corpus. (3) We created a large X corpus because the CommonCrawl corpus and the CC do not contain tweets, which would have left a significant gap in discourse coverage. Using snscrape (Python 3.11.4), we crawled and converted tweets into JSON files and stored them in CQPweb, a web-based GUI for CWB3.5 (CorpusWorkbench). We removed possible bot posts by duplicate removal and by filtering out Tweets from accounts that follow 10 users or less (i.e., although bots may have a lot of followers, they themselves tend to not follow many users). For a balanced NLP keyword approach, sampling periods of equal length are essential. We use the following key dates of the COVID-19 pandemic to define the individual sample periods: WHO Situation Report 12 (February 1, 2020), the first WHO report to mention COVID-19 on every continent, and the period between January 24 and February 5 as the period with the largest relative increase (average growth + 272% per day) in global mentions of COVID-19 in social media, establishing it as a global pandemic;^26^ the period between February 1 and March 1, 2022, as a period in which everyday COVID-19 restrictions were lifted in many countries (e.g., USA, UK, India, Canada, Israel, and the majority of EU states), resulting in a sharp reduction in interactions on social media (global average growth—25.1% per day).^27^ The X-corpora consist of 775,618,492 words, 14,320 Tweets contained the strings “palliative care” OR “palliative med*” (02/2020–02/2022: 9317; 03/2022–02/2024: 5003). Websites and X accounts of specialist societies, palliative care units, and physicians were excluded (blacklisting) in order to only represent nonprofessional discourses on the topic. As a reference sample, websites and tweets with the same strings before and after the COVID-19 pandemic were used. The University of Erlangen’s Institutional Review Board (IRB) for the Protection of Human Subjects determined data mining analyses exempt from review and oversight because due to the lack of human involvement, it does not fall under EU Regulation 536/2014 but under EU Regulation 2016/679 (GDPR). We followed the GESIS guidelines for the analysis of Twitter data (TweetsCOV19) at the Leibniz Institute for the Social Sciences in Mannheim. The processing for data analysis purposes is based on the legal provisions of Art. 6 § 1 lit. b of EU Regulation 2016/679 (GDPR). When anonymizing the data collected from individuals, especially tweets, we follow the suggestions of Mason & Singh^28^

Before conducting the study, the following steps of language data preprocessing were taken: tokenization (segmentation of the text in individual words), nonalphanumeric character removal, stop word removal (removal of irrelevant words like prepositions and conjunctions), PoS-classification (assignment of parts of speech), and Lemmatization (reducing inflected forms to a common basic form, e.g., *speak*, *speaks*, and *spoke* > *speak*).^3,5,23–26^ The following metadata was assigned to create the subcorpora: month, text type (tweet, comment, other websites), country-specific URL. Based on the corpus, we conducted an empirical analysis of the semantics and emotional associations of words, carrying out collocation calculations and semantic network analyzes.^23,25^ Frame analysis through the calculation of collocations (words that are particularly strongly associated with the search term) was based on the effect size measure log ratio confidence interval (LRC) and a 0.01% significance filter.^3,23^ In particular, the 95% CI is the range of values for parameter *θ* such that the corresponding test based on *p* value is not rejected (P(*δ*(*Y*) ≥ *δ*(***y***)|*H*_0_(*θ*)) ≥ 0.05). We then visualized the collocations in semantic networks. Texts were analyzed for their sentiment (emotional assessment of a given situation through the words used in context) using NewsMTSC, a dataset for target-based sentiment classification.^29^ Key figures between −1 and 1 indicate how strongly a word conveys a certain emotion, e.g., the word *murder* carries a strong negative sentiment (− 1), while *flower* carries a positive sentiment (+0.85). Additionally, on these “entry points into the discourse,^23^ passages of text, especially tweets, were reviewed by manual reading.

## Results

**FIG. 2.**
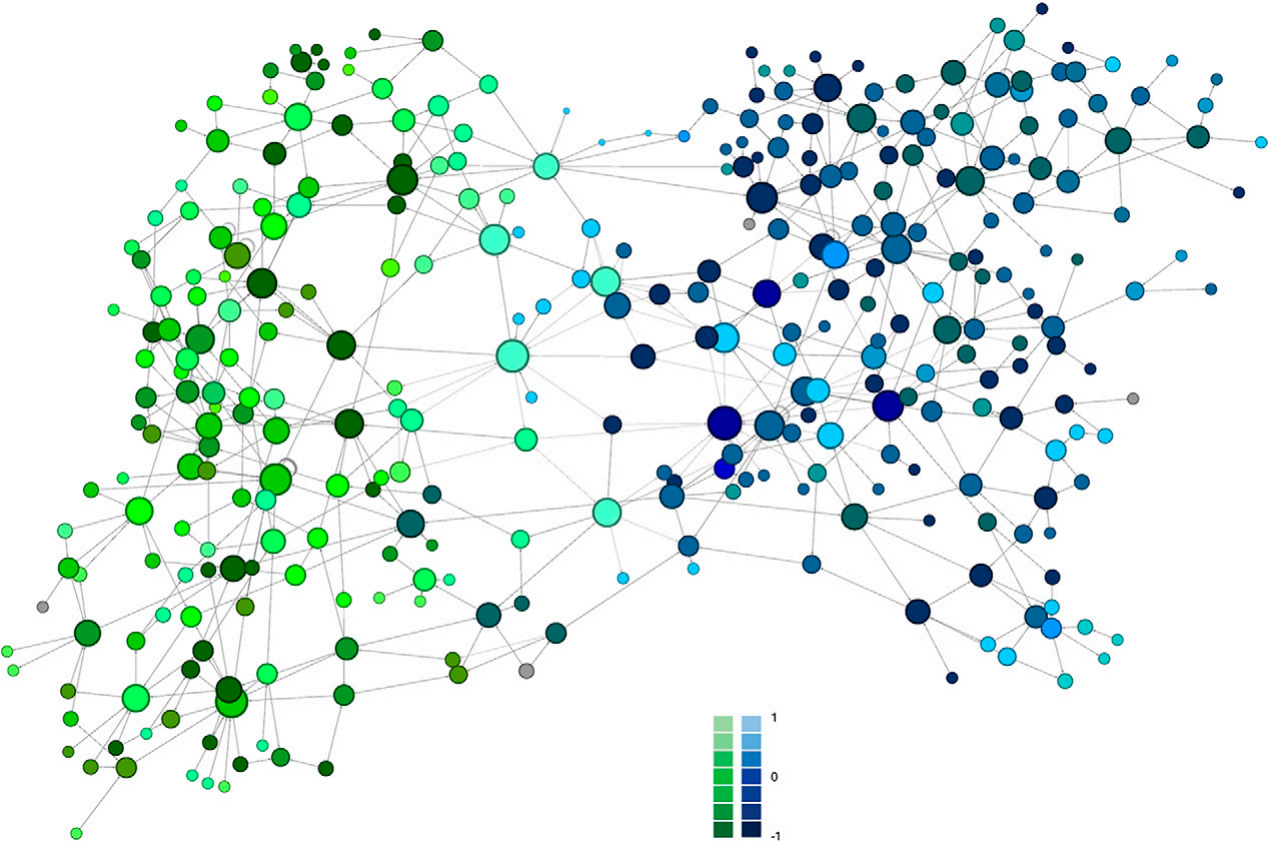
Sentiment of keywords and their collocations of the website datasets (green) and the X-datasets (blue), 4aMI2(3), 2022–2024; R5-L5, C5-NC1; stop words removed. Light colors indicate positive sentiment (1/0), dark colors indicate negative sentiment (0/−1); sizes indicate LRC effect size values. LRC, log ratio confidence interval.

Based on the corpus-linguistic analysis of the dataset, we extracted opinions and attitudes regarding the effects of the COVID-19 pandemic on palliative care. As can be seen from the structure and choice of words of the entire discourse analyzed, wording and sentiment of content on X and on websites differed significantly (Fig. 2). The pandemic and its consequences were assessed rather negatively throughout the English-language discourse between 2020 and 2024, especially because the number of negative emotion words has increased and the likelihood of their occurrence has increased.^5^ This was most clearly visible on X, where sentiment was generally more negative than in other online media (Fig. 2).

**FIG. 3.**
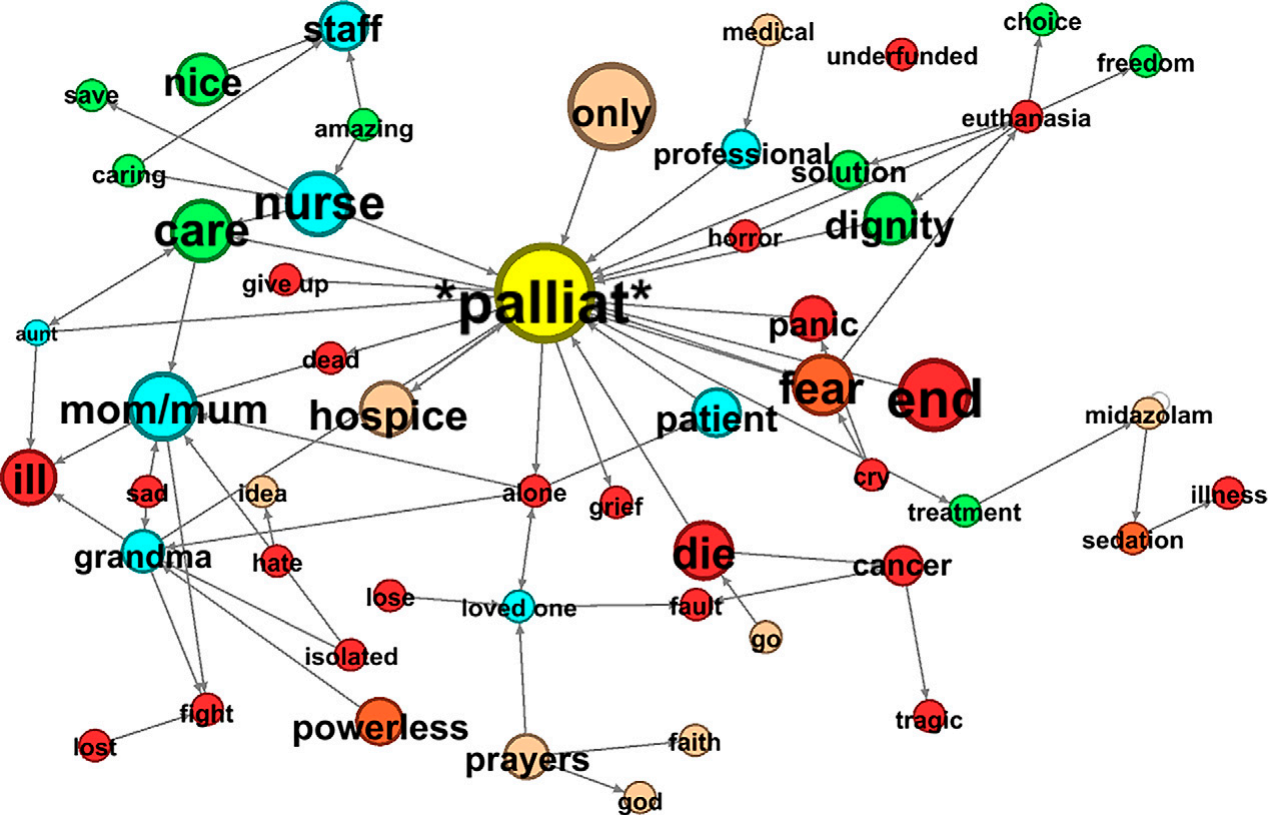
Sentiment of collocations of the node *palliat* on web platform X 2022–2024, 4a-MI2(3), R5-L5, C5-NC1; stop words removed. Green: positive sentiment (1/0), red: negative sentiment (0/−1), blue: actors; size: LRC effect size value. LRC, log ratio confidence interval.

Since 2020, users talked more often about palliative care than before the COVID-19 pandemic. Applying a minimum frequency threshold of *f* ≥10 in the Corpus, we obtained the 100 top-scoring keywords, words that are particularly important for the representation of palliative care on X, for each dataset. In a direct comparison between the study periods, it became clear that the frequency of words that negative sentiment on X in the immediate context of the strings *palliat* and *end-of-life* increased significantly (Fig. 3, Supplementary Data S1), meaning that emotion words like *fear*, *panic*, *powerless*, and *grief* were more prevalent. The strength of the negative sentiment decreased continuously from the beginning of 2022 until similar values to pre-pandemic sentiment were reached in May 2023. After the overall sentiment, we identified the most important topics from the corpus since the crisis situation of the pandemic started.

### Changing symptoms

The symptom profile of palliative care in online texts changed, but only during the COVID-19 pandemic. During the pandemic, the string *palliat* was highly associated with symptoms directly related COVID-19 (*shortness of breath*, *suffocation*, *artificial respiration*, *fear*, *restlessness*, or *panic*), while pain symptoms became less important. After the end of the pandemic, the words associated with palliative care in the collocations are similar to those before the pandemic.

### Patient welfare

Palliative situations were experienced as situations with a negative emotional content (Fig. 3, Supplementary Data S1). Psychosocial consequences of the pandemic, isolation requirements, and loneliness were recognized as a central problem.^28^ Patients and relatives reflected on the events of the pandemic, using X as a medium for situation-specific coping.^5^ As shown in Figure 3, after the COVID-19 pandemic, the transition to a palliative situation is still experienced more negatively than before and is characterized by fear and uncertainty.

### Scarcity of resources

Tweets and press releases showed a high level of awareness of the scarcity of resources since the pandemic. In press texts, questions of medical prioritization were discussed, in particular the question who should receive which kind of care. In many texts, vaccination measures for older people in palliative care settings were called into question. At the same time, the inadequate funding of palliative care was highlighted, a trend that continued in the post-pandemic data in the United Kingdom and the United States (*underfunded*, Fig. 3). After the pandemic, an accumulation of structural and political terms can be observed, including the keywords *service*, *funding*, *structures*, *NHS*, *social*, *system,* and others (SM). The issue of palliative care funding is increasingly being raised in health policy discussions.

*Fully agree with Dr Amy Proffitt, Palliative Medicine at Bart’s NHS Trust … “Its unforgivable that two-thirds of palliative care funding comes from charity.”* [TWCorp3, 21122023/2]

Similar calls for stable, independent funding of palliative care are also a part of the US discourse, for example:

*I don’t understand why they don’t want to pump more money into this.The pandemic has shown us that palliative care in the US is underfunded, but particularly needed.* [TWCorp3, 21042023]

### Challenges for medical caregivers

Frequent words such as *isolation*, *isolated*, and *alone* and their negative connotations show that the lack of social interaction as a result of isolation requirements and situations on overcrowded wards with little privacy and high workload were perceived as emotionally negative^28^ (SM). To point out the extent of the problem, metaphors of mechanics were often used, which are linked to semantic mechanisms of depersonalization. The negative evaluations did not relate to palliative care as an institution. After the pandemic, these issues no longer play a noticeable role in tweets and websites.

### Knowledge and misconceptions

As the growth rates *G* in Table 1 show, the COVID-19 pandemic promoted the spread of misconceptions.

**Table 1.** Changes in Misconceptions Occurring in Tweets Before, During, and After the Pandemic

Misconception	*n* before	*n* during	*n* after	*G*
(1) Palliative care means ending “real” treatment measures	32	37	34	+ 6,25
(2) Palliative care patients are doomed to die	38	106	42	+10,52
(3) Palliative care is sedation	45	42	41	- 9,9
(4) Palliative care is euthanasia	8	15	13	+ 62,5
(5) Palliative care means suffering	9	22	23	+ 155,5

The growth rate *G* (in percent) refers to the X datasets before and after the pandemic.

#### Ending “real” treatment measures

According to this narrative, palliative care is only used when other treatment methods no longer promise (curative) success (“patients *only* receive palliative care”). This misconception has been occurring more frequently since 2020. Palliative care is seen equivalent to end-of-life care and is structurally devalued by keywords with high effect size values such as *only*, *ending*, *stopping*, *finally*, *last*, *end*, and *death*. Apparently, for many nonprofessional participants in the discourse, receiving palliative care means giving up “real” treatment. This misconception continues to occur frequently after the end of the pandemic.

#### Patients are doomed to die

Here, the start of palliative care appears as a “death sentence.” Nonprofessional discourse actors shared the opinion that people who died of COVID-19 in a palliative treatment context should not be counted as COVID-19 deceased in statistics because they “would have died anyway.” Sometimes, the transition to a palliative care situation was presented as a direct consequence of triage decisions.

*I’m pretty sure it means “They had Covid and were dying, so they were placed in palliative care: I’m going to make that sound like they were in palliative care before they got Covid”. Grossly misleading the public in order to avoid scrutiny of aged care Covid deaths. #Liars*

#### Palliative care is sedation

In the pandemic situation, palliative care is exclusively associated with alleviating symptoms; the aim is to “accompany” people into death with sedation. Aspects of the multiprofessional approach of palliative care appear less important.

#### Palliative care is euthanasia

Since the beginning of the pandemic, palliative care has increasingly been linked to euthanasia.

*In palliative care they say we shortened the suffering of people, I call it “euthanasia”. Disgusting.* [TWCorp3, 16012024]

Despite the increase, no systematic confusion between euthanasia and palliative care was observed in the data on a large scale. Most users on X clearly distinguish palliative care from euthanasia and often act as corrective authorities for misleading claims. Since the pandemic, the distinction between palliative care and euthanasia plays a more important role on the Internet; the narrative of palliative care as an alternative to euthanasia is increasingly becoming a trademark of the discipline.

#### Palliative care means suffering

Since mid-2021, it has become more common for euthanasia advocates to frame palliative care situations with words such as *suffocate*, *blood*, *traumatic*, *nightmare*, as horrific experiences to promote their own agenda, while they are claiming positive words like *dignity*, *solution*, *choice*, *freedom,* and *autonomy* for their positions. Palliative care-related discourse positions are increasingly attacked.

*A horrific, fearful, traumatic suffering we wouldn’t see a dog endure. Palliative care is blocking this, but with Watertight legislation&safeguarding, mental health assessments & counseling we can be a progressive compassionate country.Even with the best palliative care, there is NO dignity at the end.* [TWCorp3, 16022024]

### Metaphorical use

The terms *palliative care*/*palliative medicine* are increasingly used as metaphors in everyday language. Metaphor is a special linguistic phenomenon, using one or several words to modify a target concept that is different from the source domain.^23^ Examples of metaphorical use include not only the following tweets from the US presidential election campaign and the 2024 EU parliamentary election campaign but also everyday contexts:

*Obamacare is basically palliative care, it is not health care. Every socialized medicine plan in the world is unraveling and this idiot [Biden] wants to race towards it.* [TWCorp3, 27122023]

*The election won’t change anything. The EU is on life-support and ready for palliative care unit.* [TWCorp3, 25122023/4]

*The plants in the corner of my room are in a palliative care situation.* […] [TWCorp3, 16112023/2]

In the first example, palliative care appears to be an inferior form of medicine that only relieves symptoms without addressing the actual cause. In the second tweet, the EU appears as a weak institution with no agency or prospect of improvement. The third tweet assumes that the drying houseplants will soon die. The X dataset showed a significant increase in this imagery, as no frequently occurring metaphors and palliative care-related stereotypes existed before the pandemic. Based on the collocations, this could be related to the fact that palliative care during the pandemic was more often portrayed as hopeless dying situation.

## Conclusions

In our study, we examined changes in perception, emotional attitudes, and knowledge in the international discourse on palliative care during and after the COVID-19 pandemic. Tweets and online press releases related to palliative care were statistically evaluated using a conventional NLP method.^3,22^ The awareness of palliative care has increased on the Internet because it was a relevant topic in certain phases of the COVID-19 pandemic.

### Negative emotions dominate the discourse

Sentiment analysis shows that the conditions in palliative care situations were perceived more negatively than before the pandemic.^5^ The more frequent occurrence of words with negative sentiment is associated with the fact that the pandemic was a crisis situation with scarce resources and patients facing significant stress and anxiety.^2,8^ However, the perception of the conditions in palliative care situations improved little after the pandemic, partly related to the negative framing of palliative care situations by euthanasia advocates. Our study shows that the COVID-19 pandemic has had little long-term impact on the perception of palliative care as an institution. Professional media actors and X users highlighted the value of palliative care in light of the pandemic situation. There is no lasting association with euthanasia because palliative care managed to clearly differentiate itself from this in the Internet discourse.

### Misconceptions are widespread

Our data show that entering a palliative situation is associated with more anxiety because palliative care is mostly seen as a synonym for end-of-life care. Due to misconceptions, palliative care appears to be a form of accompaniment at the end of life without any interventions apart from sedative medication. Although palliative care has been discussed more frequently in the media, we observed no systematic improvement in the knowledge of laypeople on the Internet, internalized perceptions of palliative care often are inaccurate or even misconceptions.^3,5^ Since the COVID-19 pandemic, we could observe an overall increase in the occurrence of misconceptions and negative stereotypes that had previously been known from studies.^28,29^

### Personal experiences shape beliefs

The individual understanding of palliative care, especially on X, is derived from limited *ad hoc* personal experiences focusing on the end of life and not the holistic and multidimensional palliative care approach.^5,11^

### Negative stereotypes occur more frequently

Since the beginning of the pandemic, negative stereotypical ideas about palliative approaches have been spread more frequently in metaphors and comparisons.^22^ This suggests that palliative care situations are perceived rather negatively.^5^ Negative stereotypes of palliative care are understood by the public, even if they have so far only been used by relatively few actors. With the use of metaphors, palliative care is depicted as a deficient form of medicine because it does not pursue a curative approach.

### People understand the importance of palliative care

At the same time, many other participants in the discourse recognized the importance of palliative care called for stable financing of services. Public opinions on X reflected a need for enhanced palliative care services.

### Limitations

Although we tried to capture a large section of the online discourse on the subject, reliable statements about the overall discourse are not possible.

Our study adds important insight into the long-term consequences of the COVID-19 pandemic on the perception of palliative care, which tend to be more negative perception and a higher occurrence of misconceptions regarding palliative care situations. Lack of knowledge can lead to underuse of palliative care by patients, limit informed decisions based on communication, and discourage individuals from seeking patient-centered care at the end of life. Educational interventions need to be implemented to promote knowledge of palliative care, including involvement early integration alongside life-prolonging therapies, while also directly addressing misconceptions.^30^ Overall, there is a need to increase visibility of accurate palliative care information on the Internet and to address the identified misconceptions through targeted social media campaigns.^31^ These could include educational content such as posts or infographics, interviews, hashtag campaigns as well as interactive content like Q&A sessions with palliative care experts. Another useful way of transferring information could be partnerships with social media influencers because they have a high reach and visibility, while engaging viewers on an emotional level. The use of NLP methods such as text-global sentiment analysis, keyword analysis or large language models trained on suitable data makes it possible to measure the success of such strategies with high accuracy. Social media platforms provide interesting possibilities for patients, professionals, and caregivers to share information and experiences about palliative care, making it potentially valuable for explaining palliative care approaches to the public. Policymakers should create guidelines for using social media in public health communication, ensuring the public representation of palliative care stays clear, evidence-based, and compassionate, encouraging collaboration with health care professionals and influencers, and fostering interactive engagement with to tackle misconceptions and raise awareness.

## Abbreviations Used

CC: Coronavirus Corpus
QOL: Quality of Life

## References

[1] Radbruch L, de Lima L, Knaul F, et al. Redefining palliative care—a new consensus-based definition. J Pain Symptom Manage 2020;60(4):754–764; doi: 10.1016/j.jpainsymman.2020.04.027 32387576 PMC8096724

[2] Lowe S, Pereira SM, Yardley S. Communication in palliative care during the COVID-19 pandemic: Lessons from rapidly changing, uncertain, complex, and high-stake interventions. Palliat Med 2021;35(7):1222–1224; doi: 10.1177/02692163211023208 34098795

[3] Peters J, Dykes N, Heckel M, et al. Präsentation von Palliativstationen und SAPV-Teams im Internet – eine korpusbasierte Metaanalyse von Webseiten. Z Palliativmed 2022;23(01):46–53; doi: 10.1055/a-1689-7524

[4] Pastrana T, De Lima L, Pettus K, et al. The impact of COVID-19 on palliative care workers across the world: A qualitative analysis of responses to open-ended questions. Palliat Support Care 2021;19(2):187–192; doi: 10.1017/S1478951521000298 33648620 PMC7985903

[5] Inoue M, Li MH, Hashemi M, et al. Opinion and sentiment analysis of palliative care in the era of COVID-19. Healthcare (Basel) 2023;11(6):855; doi: 10.3390/healthcare11060855 36981512 PMC10048418

[6] Wang Y, Chukwusa E, Koffman J, et al. Public opinions about palliative and end-of-life care during the COVID-19 pandemic: Twitter-based content analysis. JMIR Form Res 2023;7:e44774; doi: 10.2196/44774 37368840 PMC10408639

[7] Hodiamont F, Schatz C, Schildmann E, et al. The impact of the COVID-19 pandemic on processes, resource use and cost in palliative care. BMC Palliat Care 2023;22(1):36; doi: 10.1186/s12904-023-01151-2 37024852 PMC10077306

[8] Radbruch L, Knaul FM, de Lima L, et al. The key role of palliative care in response to the COVID-19 tsunami of suffering. Lancet 2020;395(10235):1467–1469; doi: 10.1016/S0140-6736(20)30964-8 32333842 PMC7176394

[9] Bernardis A, Gonzalez-Jaramillo V, Ebneter AS, et al. Palliative care and COVID-19: A bibliometric analysis. BMJ Support Palliat Care 2023;1; doi: 10.1136/spcare-2022-004108 36702518

[10] Cousins E, D, Vries K, H, Dening K. COVID-19 and ethical care at the end of life: Using qualitative media analysis to understand experiences of care home residents with dementia. Palliativ Med 2021;35:220–221; doi: 10.1177/0969733020976194

[11] Schwartz L, Nouvet E, de Laat S, et al. Aid when ‘there is nothing left to offer’: Experiences of palliative care and palliative care needs in humanitarian crises. PLOS Glob Public Health 2023;3(2):e0001306; doi: 10.1371/journal.pgph.0001306 36962993 PMC10021221

[12] Mitchinson L, Dowrick A, Buck C, et al. Missing the human connection: A rapid appraisal of healthcare workers’ perceptions and experiences of providing palliative care during the COVID-19 pandemic. Palliat Med 2021;35(5):852–861; doi: 10.1177/02692163211004228 33775169 PMC8114443

[13] Grant MS, Back AL, Dettmar NS. Public perceptions of advance care planning, palliative care, and hospice: A scoping review. J Palliat Med 2021;24(1):46–52; doi: 10.1089/jpm.2020.0111 32614634

[14] Parajuli J, Chen ZJ, Walsh A, et al. Knowledge, beliefs, and misconceptions about palliative care among older adults with cancer. J Geriatr Oncol 2023;14(1):101378; doi: 10.1016/j.jgo.2022.09.007 36182659

[15] Metin S, Demirci H, Metin AT. Effect of health literacy of caregivers on survival rates of patients under palliative care. Scand J Caring Sci 2019;33(3):669–676; doi: 10.1111/scs.12662 30735265

[16] McIlfatrick S, Slater P, Beck E, et al. Examining public knowledge, attitudes and perceptions towards palliative care: A mixed method sequential study. BMC Palliat Care 2021;20(1):44; doi: 10.1186/s12904-021-00730-5 33731087 PMC7971949

[17] Mallon A, Slater P, Hasson F, et al. What do young adults know about palliative care? A cross-sectional survey. Public Health 2021;191:78–84; doi: 10.1016/j.puhe.2020.11.023 33545498

[18] Fishman JM, Greenberg P, Bagga MB, et al. Increasing Information Dissemination in Cancer Communication: Effects of Using “Palliative,” “Supportive,” or “Hospice” Care Terminology. J Palliat Med 2018;21(6):820–824; doi: 10.1089/jpm.2017.0650 29676957

[19] Iwendi C, Mohan S, Khan S, et al. Covid-19 fake news sentiment analysis. Comput Electr Eng 2022;101:107967; doi: 10.1016/j.compeleceng.2022.107967 35474674 PMC9023343

[20] Selman LE, Chamberlain C, Sowden R, et al. Sadness, despair and anger when a patient dies alone from COVID-19: A thematic content analysis of Twitter data from bereaved family members and friends. Palliat Med 2021;35(7):1267–1276; doi: 10.1177/02692163211017026 34016005 PMC8267082

[21] Baranowski AM, Blank R, Maus K, et al. ‘We are all in the same boat’: A qualitative cross-sectional analysis of COVID-19 pandemic imagery in scientific literature and its use for people working in the German healthcare sector. Front Psychiatry, 2024;15(15):1296613; doi: 10.3389/fpsyt.2024.1296613 38374972 PMC10875073

[22] Sarmet M, Kabani A, Coelho L, et al. The use of natural language processing in palliative care research: A scoping review. Palliat Med 2023;37(2):275–290; doi: 10.1177/02692163221141969 36495082

[23] Baker P. Using Corpora in Discourse Analysis. Bloomsbury: London; 2023.

[24] Davies M. The Coronavirus Corpus Design, construction, and use. IJCL 2021;26(4):583–598; doi: 10.1075/ijcl.21044.dav

[25] Panchenko A, Ruppert E, Faralli S, et al. Building a Web-Scale Dependency-Parsed Corpus from CommonCrawl. In: ACL Anthology; 2018. Available from: https://aclanthology.org/L18-1286 [last accessed 06/24/2024].

[26] Cinelli M, Quattrociocchi W, Galeazzi A, et al. The COVID-19 social media infodemic. Sci Rep 2020;10(1):16598; doi: 10.1038/s41598-020-73510-5 33024152 PMC7538912

[27] Dimitrov D, Baran E, Fafalios, et al. TweetsCOV19 – A Knowledge Base of Semantically Annotated Tweets about the COVID-19 Pandemic, (CIKM2022), Resource Track, ACM 2023.

[28] Mason S, Singh L. Reporting and discoverability of “Tweets” quoted in published scholarship: Current practice and ethical implications. Research Ethics 2022;18(2):93–113; doi: 10.1177/17470161221076948

[29] Hamborg F, Donnay K. NewsMTSC: A Dataset for (Multi-)Target-dependent Sentiment Classification in Political News Articles. In: Proceedings of the 16th Conference of the European Chapter of the Association for Computational Linguistics: Main Volume 2021;1:1663–1675; doi: 10.18653/v1/2021.eacl-main.142

[30] Fliedner, M. C., Zambrano, S. C., & Eychmueller, S. Public perception of palliative care: A survey of the general population. Palliat Care Soc Pract. 2021;15;15:26323524211017546; doi: 10.1177/26323524211017546 34164622 PMC8191057

[31] Easwar S, Alonzi S, Hirsch J, et al. Palliative care tiktok: Describing the landscape and explaining social media engagement. J Palliat Med 2023;26(3):360–365; doi: 10.1089/jpm.2022.0250 36112152

