# Supplementary material for: What Does “Palliative” Mean? Sentiment, Knowledge, and Public Perception Concerning Palliative Care on the Internet since the COVID-19 Pandemic
Source: Palliat Med Rep. 2024 Dec 4;5(1):512–20. doi: 10.1089/pmr.2024.0057 (PMC11693953; doi:10.1089/pmr.2024.0057)
Supplement: Supplementary Data S1 [file pmr.2024.0057_supp_datas1.docx]

**Supplementary material**

| **#** | **Word** | **Freq.** | **Exp. Freq.** | **Log ratio** |
| --- | --- | --- | --- | --- |
| 1 | end of life | 522 | 0.528 | 15.385 |
| 2 | grief | 174 | 0.869 | 14.366 |
| 3 | restriction | 188 | 0.123 | 14.144 |
| 4 | death | 48 | 0.031 | 14.092 |
| 5 | hospice | 376 | 0.246 | 13.906 |
| 6 | alleviate | 150 | 0.098 | 13.705 |
| 7 | hospice | 646 | 0.096 | 13.457 |
| 8 | fear | 191 | 0.125 | 13.471 |
| 9 | funeral | 254 | 0.166 | 13.362 |
| 10 | training | 149 | 0.098 | 13.348 |
| 11 | voluntarily | 1724 | 0.148 | 13.313 |
| 12 | medical | 1507 | 0.148 | 13.261 |
| 13 | nurse | 398 | 0.142 | 13.206 |
| 14 | sedate | 25 | 0.137 | 13.15 |
| 15 | relative | 24 | 0.131 | 13.091 |
| 16 | pediatric | 22 | 0.12 | 12.965 |
| 17 | restriction | 19 | 0.104 | 12.754 |
| 18 | outpatient | 2752 | 4.799 | 12.676 |
| 19 | pain relieving | 16 | 0.088 | 12.676 |
| 20 | terminally | 16 | 0.088 | 12.506 |
| 21 | advice | 3572 | 2.336 | 6.085 |
| 22 | cancer | 1192 | 0.78 | 5.906 |
| 23 | die | 569 | 0.372 | 5.872 |
| 24 | ill | 945 | 0.618 | 5.376 |
| 25 | priest | 795 | 0.52 | 5.373 |
| 26 | forget | 122 | 3.92 | 1.513 |
| 27 | laugh | 27 | 0.121 | 1.473 |
| 28 | factor | 52 | 0.434 | 1.399 |
| 29 | let | 365 | 1.24 | 1.289 |
| 30 | lose | 33 | 0.235 | 1.204 |

**Appendix 1:** 30 most important collocations of the string palliative in the web corpus, log ratio (filtered), minimum frequency 15, range: 5L, 5R; 2018-2020.

| **#** | **Word** | **Freq.** | **Exp. Freq.** | **Log ratio** |
| --- | --- | --- | --- | --- |
| 1 | opiate | 160 | 0.328 | 14.413 |
| 2 | COVID | 104 | 0.569 | 14.192 |
| 3 | alleviate | 50 | 0.274 | 14.15 |
| 4 | end of life | 50 | 0.274 | 14.15 |
| 5 | crisis | 47 | 0.257 | 14.6 |
| 6 | restrictions | 34 | 0.186 | 13.593 |
| 7 | hospice | 1682 | 9.123 | 13.556 |
| 8 | fear | 61 | 0.334 | 13.413 |
| 9 | patient | 120 | 0.657 | 13.388 |
| 10 | infected | 119 | 0.651 | 13.376 |
| 11 | lonely | 27 | 0.148 | 13.313 |
| 12 | visitation | 27 | 0.148 | 13.261 |
| 13 | ward | 26 | 0.142 | 13.206 |
| 14 | sedate | 25 | 0.137 | 13.15 |
| 15 | infection | 24 | 0.131 | 13.091 |
| 16 | children | 22 | 0.12 | 12.965 |
| 17 | isolation | 19 | 0.104 | 12.754 |
| 18 | punishable | 18 | 0.099 | 12.676 |
| 19 | pain | 16 | 0.088 | 12.676 |
| 20 | terminally | 16 | 0.088 | 12.506 |
| 21 | relieve | 16 | 0.088 | 12.506 |
| 22 | ward | 27 | 0.148 | 12.206 |
| 23 | painkiller | 22 | 0.12 | 11.898 |
| 24 | burden | 73 | 0.4 | 11.614 |
| 25 | isolation | 16 | 0.088 | 11.413 |
| 26 | shortness | 22 | 0.092 | 11.413 |
| 27 | panic | 27 | 0.101 | 11.403 |
| 28 | breath | 52 | 0.134 | 11.399 |
| 29 | see | 3685 | 352.4 | 11.389 |
| 30 | choke | 43 | 0.235 | 11.304 |

**Appendix 2:** 30 most important collocations of the string palliative in the web corpus, log ratio (filtered), minimum frequency 15, range: 5L, 5R; 2020-2023.

| **#** | **Word** | **Freq.** | **Exp. Freq.** | **Log ratio** |
| --- | --- | --- | --- | --- |
| 1 | end of life | 612 | 0.528 | 15.355 |
| 2 | patient | 154 | 0.869 | 13.364 |
| 3 | death | 187 | 0.123 | 10.143 |
| 4 | ward | 64 | 0.031 | 10.075 |
| 5 | hospice | 364 | 0.246 | 9.909 |
| 6 | burden | 153 | 0.098 | 9.699 |
| 7 | funding | 646 | 0.096 | 9.443 |
| 8 | grief | 131 | 0.125 | 8.478 |
| 9 | funeral | 264 | 0.166 | 8.333 |
| 10 | training | 149 | 0.098 | 7.885 |
| 11 | medicine | 1594 | 0.148 | 6.313 |
| 12 | doctor | 1007 | 0.148 | 6.261 |
| 13 | nurse | 448 | 0.142 | 6.206 |
| 14 | pain | 65 | 0.137 | 6.15 |
| 15 | relative | 24 | 0.131 | 6.091 |
| 16 | pediatric | 22 | 0.12 | 5.965 |
| 17 | restriction | 19 | 0.104 | 5.754 |
| 18 | relative | 952 | 4.799 | 5.699 |
| 19 | midazolam | 18 | 0.088 | 5.676 |
| 20 | brighten | 18 | 0.088 | 5.676 |
| 21 | they | 3272 | 2.336 | 5.085 |
| 22 | cancer | 1400 | 0.78 | 4.906 |
| 23 | die | 509 | 0.372 | 4.872 |
| 24 | ill | 915 | 0.618 | 4.376 |
| 25 | spiritual | 788 | 0.52 | 4.373 |
| 26 | anxiety | 101 | 3.92 | 3.513 |
| 27 | laugh | 29 | 0.121 | 3.473 |
| 28 | success | 47 | 0.434 | 3.399 |
| 29 | lose | 372 | 1.24 | 3.289 |
| 30 | depression | 34 | 0.235 | 3.204 |

**Appendix 3:** 30 most important collocations of the string palliative in the web corpus, log ratio (filtered), minimum frequency 15, range: 5L, 5R; 2022-2024.

| **#** | **Word** | **Freq.** | **Exp. Freq.** | **Log ratio** |
| --- | --- | --- | --- | --- |
| 1 | cancer | 1006 | 55.3 | 19.523 |
| 2 | care | 432 | 85.088 | 13.506 |
| 3 | staff | 27 | 0.148 | 12.206 |
| 4 | mom | 22 | 0.12 | 11.898 |
| 5 | nurse | 73 | 0.4 | 11.614 |
| 6 | treatment | 16 | 0.088 | 11.413 |
| 7 | lose | 41 | 0.224 | 11.169 |
| 8 | dignitiy | 811 | 0.607 | 11.008 |
| 9 | die | 204 | 1.116 | 10.965 |
| 10 | lose | 23 | 0.126 | 10.898 |
| 11 | hope | 77 | 0.421 | 10.828 |
| 12 | support | 250 | 1.368 | 10.552 |
| 13 | ill | 17 | 0.093 | 10.413 |
| 14 | hospice | 38 | 0.208 | 10.228 |
| 15 | ill | 29 | 0.159 | 10.215 |
| 16 | friendly | 58 | 0.317 | 10.194 |
| 17 | illness | 86 | 0.197 | 10.138 |
| 18 | patient | 70 | 0.383 | 10.091 |
| 19 | medicine | 62 | 0.339 | 10.064 |
| 20 | experience | 40 | 0.219 | 10.008 |
| 21 | dear | 72 | 0.394 | 9.828 |
| 22 | solution | 42 | 0.23 | 9.828 |
| 23 | nice | 41 | 0.224 | 9.786 |
| 24 | competent | 41 | 0.224 | 9.786 |
| 25 | grandma | 236 | 1.292 | 9.631 |
| 26 | panic | 22 | 0.092 | 9.413 |
| 27 | broken | 27 | 0.101 | 9.403 |
| 28 | alleviate | 52 | 0.134 | 9.369 |
| 29 | it | 13644 | 1332.2 | 5.329 |
| 30 | go | 1143 | 7.235 | 4.304 |

**Appendix 4:** 30 most important collocations of the string palliative in the Twitter corpus, log ratio (filtered), minimum frequency 15, range: 5L, 5R; 2018-2020.

| **#** | **Word** | **Freq.** | **Exp. Freq.** | **Log ratio** |
| --- | --- | --- | --- | --- |
| 1 | only | 990 | 55.3 | 17.332 |
| 2 | terminal | 114 | 1.088 | 15.25 |
| 3 | limitation | 454 | 0.27 | 14.162 |
| 4 | die | 454 | 0.27 | 13.172 |
| 5 | end | 150 | 1.253 | 13.082 |
| 6 | hospice | 144 | 1.183 | 13.015 |
| 7 | coronavirus | 691 | 9.065 | 12.878 |
| 8 | COVID | 165 | 0.329 | 12.735 |
| 9 | mom | 124 | 0.647 | 12.511 |
| 10 | horrible | 116 | 0.641 | 12.498 |
| 11 | fear | 30 | 0.146 | 12.365 |
| 12 | cancer | 230 | 0.346 | 12.183 |
| 13 | final | 206 | 0.34 | 12.128 |
| 14 | grandma | 29 | 0.135 | 12.072 |
| 15 | isolation | 25 | 0.129 | 12.013 |
| 16 | overload | 24 | 0.119 | 11.787 |
| 17 | infected | 19 | 0.102 | 11.776 |
| 18 | suffocate | 18 | 0.097 | 11.698 |
| 19 | powerless | 18 | 0.097 | 11.698 |
| 20 | sedation | 157 | 5.086 | 10.528 |
| 21 | leave | 17 | 0.086 | 10.528 |
| 22 | isolated | 57 | 1.046 | 10.228 |
| 23 | dead | 22 | 0.119 | 9.822 |
| 24 | angry | 69 | 0.393 | 9.606 |
| 25 | vaccinate | 129 | 0.186 | 9.43 |
| 26 | treatment | 55 | 1.086 | 9.425 |
| 27 | grandfather | 257 | 3.07 | 9.366 |
| 28 | professional | 44 | 2.049 | 9.345 |
| 29 | alone | 353 | 5.393 | 9.328 |
| 30 | patient | 186 | 8.079 | 9.3 |

**Appendix 5:** 30 most important collocations of the string palliative in the Twitter corpus, log ratio (filtered), minimum frequency 15, range: 5L, 5R; 2020-2022.

| **#** | **Word** | **Freq.** | **Exp. Freq.** | **Log ratio** |
| --- | --- | --- | --- | --- |
| 1 | only | 1240 | 55.3 | 18.132 |
| 2 | end | 104 | 85.088 | 15.21 |
| 3 | hospice | 450 | 0.27 | 14.142 |
| 4 | mom | 450 | 0.27 | 14.142 |
| 5 | nurse | 147 | 1.253 | 14.085 |
| 6 | care | 134 | 1.183 | 13.715 |
| 7 | dignity | 682 | 9.065 | 13.678 |
| 8 | fear | 161 | 0.329 | 13.485 |
| 9 | nice | 120 | 0.647 | 13.411 |
| 10 | staff | 119 | 0.641 | 13.378 |
| 11 | ill | 27 | 0.146 | 13.235 |
| 12 | cancer | 227 | 0.346 | 13.213 |
| 13 | die | 216 | 0.34 | 13.208 |
| 14 | powerless | 25 | 0.135 | 13.172 |
| 15 | prayers | 24 | 0.129 | 13.113 |
| 16 | save | 22 | 0.119 | 12.987 |
| 17 | sad | 19 | 0.102 | 12.776 |
| 18 | fight | 180 | 0.97 | 11.698 |
| 19 | euthanasia | 18 | 0.097 | 11.698 |
| 20 | dead | 16 | 0.086 | 11.528 |
| 21 | give up | 16 | 0.086 | 11.528 |
| 22 | professional | 27 | 0.146 | 11.228 |
| 23 | solution | 22 | 0.119 | 10.622 |
| 24 | grief | 73 | 0.393 | 10.616 |
| 25 | panic | 126 | 0.186 | 10.435 |
| 26 | sedation | 16 | 0.086 | 9.422 |
| 27 | medical | 227 | 2.07 | 9.336 |
| 28 | underfunded | 12 | 0.049 | 9.315 |
| 29 | hate | 73 | 0.393 | 9.322 |
| 30 | alone | 86 | 0.079 | 8.9 |

**Appendix 6:** 30 most important collocations of the string palliative in the Twitter corpus, log ratio (filtered), minimum frequency 15, range: 5L, 5R; 2022-2024.
